# Epitope Detection in Monocytes (EDIM) As a New Method of Liquid Biopsy in Pediatric Rhabdomyosarcoma

**DOI:** 10.3390/biomedicines10081812

**Published:** 2022-07-28

**Authors:** Cristian Urla, Matias Julian Stagno, Andreas Schmidt, Rupert Handgretinger, Jörg Fuchs, Steven W. Warmann, Evi Schmid

**Affiliations:** 1 Department of Pediatric Surgery and Pediatric Urology, University Children’s Hospital of Tuebingen, 72076 Tuebingen, Germany; cristian.urla@med.uni-tuebingen.de (C.U.); matias.stagno@outlook.de (M.J.S.); andreas.schmidt@med.uni-tuebingen.de (A.S.); joerg.fuchs@med.uni-tuebingen.de (J.F.); steven.warmann@med.uni-tuebingen.de (S.W.W.); 2 Department of Pediatric Hematology and Oncology, University Children’s Hospital of Tuebingen, 72076 Tuebingen, Germany; rupert.handgretinger@med.uni-tuebingen.de

**Keywords:** EDIM, Apo10, TKTL1, pediatric rhabdomyosarcoma

## Abstract

Biomarkers allowing characterization of pediatric rhabdomyosarcoma (RMS) are lacking. Epitope detection in monocytes (EDIM) is a novel method focused on detection of the biomarkers TKTL1 (transketolase-like protein 1) and Apo10 (epitope of DNaseX) in activated monocytes (CD14^+^/CD16^+^) from patient’s blood. We investigated the expression of these biomarkers in RMS cell lines, tumor material, and peripheral blood from RMS patients. Expression levels of TKTL1 and DNaseX/Apo10 in RMS cell lines (RH30, RD) and tumor samples were analyzed by RT-PCR and flow cytometry. Blood samples of 29 RMS patients were measured and compared to 27 healthy individuals. The percentages of activated CD14^+^/CD16^+^ monocytes harboring TKTL1 and Apo10 were determined. EDIM-TKTL1 and EDIM-Apo10 expression scores were calculated. The relationship between TKTL1 expression and DNA-hypomethylation was evaluated. Both RMS cell lines and tumor samples showed significantly higher expression levels of TKTL1 and DNaseX/Apo10 compared to skeletal muscle cells (SkMC). EDIM-TKTL1 and EDIM-Apo10 scores were positive in 96.5% of patients with RMS. All healthy controls had negative corresponding scores. RMS cell lines show increased expression levels of the biomarkers TKTL1 and DNaseX/Apo10. The sensitivity of the EDIM blood test indicates that this assay might serve as an additional tool in pediatric RMS.

## 1. Introduction

Rhabdomyosarcoma (RMS) is the most common pediatric soft tissue sarcoma and the third most common extracranial solid tumor of childhood, accounting for approximately 5% of all pediatric cancers [1,2]. Despite aggressive multimodal treatment, the prognosis of children with advanced stages of the disease remains very poor, with only 25% of them expected to be free of disease 3 years after diagnosis [3]. 

Recurrences are common in patients with RMS, especially in those with alveolar histology (ARMS). Dantonello et al., analyzing the survival following disease recurrence of primary localized ARMS, found that relapses occurred in approximately 48% of the patients; they reported a survival rate of 21% on a 5-year post-relapse [4]. 

Early identification of tumor relapse may lead to an earlier initiation of therapy with possible improvement of survival [5]. Serial measurements of tumor markers or biomarkers represent a reliable method to monitor the response to therapy [6]. 

Tumor markers have been established for certain types of malignancies, such as colorectal, prostate, and breast tumors [7]. However, new tumor markers with high sensitivity and specificity are required, especially for those tumors for which no general tumor markers or biomarkers are available, as is the case for pediatric RMS [7]. 

Epitope detection in monocytes (EDIM) is a novel method for detection of biomarkers in activated monocytes (CD14^+^/CD16^+^) in the blood [8,9,10,11]. These monocytes phagocytize tumor cells and store tumor proteins intracellularly. They can be detected in samples from peripheral blood of the patients using specific antibodies [12]. Particularly, the EDIM blood test focuses on the detection of two biomarkers, TKTL1 (transketolase-like protein 1) and Apo10 (epitope of DNaseX overexpressed in cancer cells), in CD14^+^/CD16^+^ activated monocytes [12]. 

TKTL1 is an enzyme activated by the promotor DNA hypomethylation [13,14], which plays a key role in the non-oxidative arm of the pentose phosphate pathway (PPP) leading to an increased production of lactate, acidification of the tumor microenvironment, and degradation of the stroma, thus facilitating the cell migration and the development of metastasis [15]. Furthermore, TKTL1 causes tumor metabolic reprogramming and orchestrates aerobic glycolysis, fatty acid and nucleic acid synthesis, glutamine metabolism, protection against oxidative stress, and cell proliferation [16]. Recently, Li et al. have shown that TKTL1 controls the cell cycle [17]. During the cell cycle, TKTL1 is overexpressed in the late G1 phase and S phases and forms stable heterodimers with transketolase (TKT). This heterodimer formation (TKT/TKTL1) shifts transketolase activity towards ribose-5-phosphate (R5P) accumulation in the cell as a building block for new DNA, which allows rapid DNA synthesis and repair [17]. In line with the crucial and extremely “beneficial” function of TKTL1 for tumor cell proliferation, invasive growth, and metastasis, it has already been demonstrated that TKTL1 expression is associated with poor prognosis and therapy resistance in patients suffering from different types of cancer [18,19,20,21,22]. This increased expression seems to be related to the hypomethylation of the TKTL1 promoter [13]. 

The biomarker Apo10 is an epitope on the DNaseX (Desoxyribonuclease X), an endonuclease cleaving DNA in 300 bp fragments and thereby executing the final step of apoptosis, and is highly specifically overexpressed in tumor cells irrespective of the tumor entity. Apparently, tumor cells are able to inhibit the DNaseX endonuclease activity, and, as a result, the Apo10 epitope accumulates in the tumor cells nucleus, indicative of an inhibited apoptosis. Apo10 overexpression represents a very early event during malignant transformation from normal cells to tumor cells, and, therefore, might possibly be a useful biomarker for early detection of neoplasia [7]. The detection of Apo10 in CD14^+^/CD16^+^ monocytes above a certain threshold indicates phagocytosis of tumor compounds [7]. It has been shown that Apo10 is highly expressed in neoplastic cells, including in carcinomas, sarcomas, glioblastomas, lymphomas, and leukemias [7,10]. In contrast, absent or only weak expression has been observed in hematopoietic cells [12,23].

Liquid biopsy might have a potential relevance for molecular diagnosis, therapy monitoring, and follow-up in patients with RMS. Additionally, pediatric patients might benefit from the less-invasive nature of the new diagnostic methods [24].

There are no reports in the literature regarding the role of the EDIM blood test for detection of TKTL1 and Apo10 in pediatric RMS. The aim of the present study was to investigate if the abovementioned biomarkers can be detected in pediatric RMS cell lines and in tumor material, as well as in peripheral blood samples of children with RMS. Additionally, we investigated whether TKTL1 expression is associated with hypomethylation of TKTL1 promoter in pediatric RMS. 

## 2. Materials and Methods

### 2.1. Cell Lines and Culture Conditions

To study the expression of TKTL1 and Apo10 in rhabdomyosarcoma cell lines, an embryonal (RD, (RRID: CVCL_0041) ATCC, Manassas, VA, USA) and an alveolar (RH30, (RRID: CVCL_1649) DSMZ, Braunschweig, Germany) RMS cell line was used. Skeletal muscle cells (SkMC, Sigma Aldrich, Taufkirchen, Germany) were used as the control for expression. Cancer cell lines were obtained directly from a cell bank (ATCC, DSMZ; date of purchase: August 13th, 2020; PO No.: 4502127595) that performs cell line characterizations and were processed in our laboratory fewer than 6 months after receipt. 

All cells were cultured in the DMEM medium (with high glucose, sodium bicarbonate, and L-glutamine; GIBCO, Berlin, Germany) supplemented with 10% fetal calf serum (FCS; Sigma Aldrich, Taufkirchen, Germany), and antibiotics (Penicillin/Streptomycin, Biochrom, Berlin, Germany) in a humidified atmosphere containing 5% CO_2_ at 37 °C. All cells were tested mycoplasma negative (Mycoalert, Lonza, Basel, Switzerland). 

### 2.2. Immunohistochemistry

Immunohistochemistry of TKTL1 was performed according to a previously described method [25]. After demasking the epitopes, the paraffin sections from RMS tumor and muscle tissue were blocked with 3% goat serum (Dako, Agilent Technologies Inc., Santa Clara, CA, USA) and incubated over night at 4 °C with anti TKTL1 mouse monoclonal antibody JFC12T10 (1.3 mg/mL; Zyagnum AG, Pfungstadt, Germany). The following day, the Biotin-SP conjugated AffiniPure F(ab)2 fragment donkey anti-mouse IgG (1:500; Jackson Immuno Research) antibody was incubated for 45 min at room temperature. The antibody binding was detected using VECTASTAIN Universal Elite ABC kit (LINARIS GmbH, Dossenheim, Germany) and DAB solution (Dako, Agilent Technologies Inc, Santa Clara, CA, USA). The slides containing the respective tumor sections were then analyzed using a transmitted light Zeiss Axioskop 40 microscope (Carl Zeiss AG, Oberkochen, Germany, original magnification, 20×).

### 2.3. Immunofluorescence Microscopy

For immunofluorescence microscopy RMS cells and SKMC cells were grown on glass chamber slides (Sarstedt, Nümbrecht, Germany), washed three times with PBS, and fixed with ice-cold Acetone/Methanol (1:1) for 15 min at −20 °C. Subsequently the cells were washed three times with PBS with 0.2 % Tween (PBS-T), incubated for 1h at room temperature (RT) in blocking buffer containing 3% goat serum (in PBS-T; abcam, Cambridge, UK) and exposed for 30 min at RT with anti-TKTL1 mouse monoclonal antibody JFC12T10 (1:150, Zyagnum AG, Pfungstadt, Germany) or anti-DNaseX/Apo10 rat monoclonal antibody (1:50, Zyagnum AG, Pfungstadt, Germany). After three washing steps with PBS-T the cells were incubated for TKTL1 with Alexa Fluor 635 anti-mouse (1:500) and for Apo10 with Alexa Fluor 488 anti-rat (1:750) secondary antibodies (cell signaling, Cambridge, UK) for 1 h at RT. Following three washes with PBS-T all slides were covered with covering medium including DAPI (Invitrogen, Darmstadt, Germany) and images were taken on a Zeiss Apotome (Carl Zeiss Micro Imaging) with an A-Plan 40× ocular. 

### 2.4. Preparation of Tumor Material

Five RMS tumor samples (ERMS (embryonal RMS) n = 3; ARMS (alveolar RMS) n = 2) were analyzed for the mRNA expression of TKTL1 and DNaseX/Apo10. After thawing, the tumor samples were mechanically homogenized, lysed, and subjected to total RNA extraction. The transcriptional mRNA expression was determined by reverse transcription and quantitative real-time PCR. Normal muscle tissue was used as reference. 

### 2.5. RNA Isolation and Real Time PCR (qRT-PCR)

RT-PCR was used to determine the transcript levels of TKTL1 and DNaseX/Apo10. For this purpose, total RNA was extracted from RD, RH30, and SkMC cells using the RNeasy Mini Kit (Qiagen, Hilden, Germany). Reverse transcription of total RNA was performed using the high-capacity cDNA Reverse Transcription Kit (Applied Biosystems, Waltham, Massachusetts, USA). Polymerase chain reaction (PCR) amplification of each gene was performed in a total volume of 20 μL using 40 ng cDNA, 500 nM forward and reverse primers, and 2x GoTaq^®^ qPCR Master Mix (Promega Corporation, Madison, WI, USA). All kits were performed according to the manufacturer’s instructions. Cycling conditions were as follows: initial denaturation at 95 °C for 5 min, followed by 40 cycles at 57 °C for 30 s and at 72 °C for 20 s. For the amplification, the following primers were used (5′-3′orientaion):

TKTL1 fw: CGCCGAGCACTGCATAAA;
TKTL1 rev: CCACATAAGTGTTCCACCCAA A;
DNaseX/Apo10 fw: AGCTGGTGTCTGTGAAGAGG;
DNaseX/Apo10 rev: CCGTGTAGACCTCAACCAAC;
TBP fw: GCC CGA AAC GCC GAA TAT;
TBP rev: CCG TGG TTC GTG GCT CTC.

Melting curve analysis confirmed the specificity of the PCR product. Real-time PCRs were performed using a CFX96 Real-Time System (Bio-Rad, Feldkirchen, Germany) in duplicates. Amplification of the housekeeping gene TBP (TATA binding protein) was performed to standardize the amount of sample RNA. Relative quantification of gene expression was performed using the ΔCt method.

### 2.6. Flow Cytometry—Spike Experiments

To prove that CD14^+^/CD16^+^ monocytes phagocytose tumor cells and internalize them, co-culture experiments were performed. RH30 and RD cells were seeded at a density of 1 × 10^4^ cells/100 µL into 96-well flat-bottomed microtiter plates at 37 °C and 5% CO_2_. On the next day, 100 µL freshly drawn EDTA blood from healthy individuals was added either to RMS cells, to DMEM medium without cells, or to empty wells. After co-culturing for 24h in a humidified atmosphere containing 5% CO_2_ at 37 °C, the cells were harvested and analyzed through flow cytometry using an EDIM blood test. CD14^+^/CD16^+^ activated monocytes were analyzed for intracellular TKTL1 and Apo10 protein abundances. 

### 2.7. Patient’s Characteristics

The patients with histologically confirmed diagnosis of RMS, who have been treated in our department between 2015 and 2018, who were ≤21 years old, and had no previous treatment for sarcoma were included in the study. 

In total, 27 matched healthy patients were included in the study as the control group in order to determine the EDIM-TKTL1 and EDIM-Apo10 scores in healthy individuals. In the control group, participants between 0 and 18 years of age without oncological or pathological background were included. Written informed consent to participate in the study was obtained from the patients, guardians, or parents. All procedures performed were in accordance with the ethical standards of the institutional and/or national research committee and with the 1964 Helsinki declaration and its later amendments or comparable ethical standards. The study protocol was approved by the ethical committee of the Medical Faculty Tuebingen (615/2015B02 and 190/2017B01). 

### 2.8. Blood Samples

Blood samples from patients with and without rhabdomyosarcoma were collected in 2.7 mL EDTA tubes, then pseudonymized and processed within 24 h. Clinical data were blinded. Cell counting was performed using the ADVIA 120 hematology system (Siemens, Erlangen, Germany). Finally, flow cytometric analysis of the whole blood samples was performed.

### 2.9. Flow Cytometry Measurements—EDIM Blood Test

Flow cytometry measurements were performed as previously described [12]. Samples were analyzed using FACS Canto II (BD Biosciences, Heidelberg, Germany). In total, 1000 monocytes (CD14^+^/CD16^+^) from the blood sample were counted and analyzed. The analyses were performed using FACS Diva software version 8.0 (BD Biosciences, Heidelberg, Germany). The proportion of CD14^+^/CD16^+^ cells containing Apo10 and TKTL1 was multiplied by 10 to create the EDIM score, as previously described by Saman et al. [12].

### 2.10. Evaluation of Correlation between TKTL1 Expression and DNA-Hypomethylation in RMS Cells

The RH30 and RD cell lines (2 × 10^5^ cells, respectively) were treated for 72 h with 100 nM and 500 nM of DNA-methyltransferase inhibitor 5-Aza-2′-deoxycytidin (5-Aza-dC; Merck, Taufkirchen, Germany) in 6-well plates. The medium and the treatment were renewed every 24 h. After treatment, the cells were harvested, the RNA was isolated and expression levels of the TKTL1 gene were quantified using qRT-PCR, as described above. 

### 2.11. In Silico Analysis of the TKTL1 Promoter for Identification of CpG Islands

An in silico approach was chosen to screen the promoter for the presence of CpG islands. The transcription-start (TSS) of the TKTL1 gene was identified. The MethPrimer program was used to search for CpG islands in the TSS region. The CpG islands, downstream of the TSS, were examined for hypomethylation in subsequent experiments using bisulfite sequencing.

### 2.12. Bisulfite Sequencing (BS) of the TKTL1 Promoter in Tumor Material from RMS Patients

DNA from cell lines and primary tumors were subjected to bisulfite treatment, as described previously [26,27]. DNA from fibroblasts served as control. Tissue samples used for bisulfite sequencing were the same as those used for evaluation of TKTL1 expression. 

For bisulfite treatment, 2 µg of genomic DNA was denatured in a 0.2 mol/L NaOH solution at 50 °C for 20 min and diluted in 500 µL of a freshly prepared solution of 10 mmol/L hydroquinone and 3 mol/L sodium bisulfite. After incubation at 70 °C for 3 h, the DNA sample was desalted over a column (Wizard DNA Clean-Up System; Promega, Madison, WII, USA), treated with 0.3 mol/L NaOH for 10 min at room temperature, and precipitated overnight with ethanol. 

The region of interest was amplified with specific primers via touchdown PCR (TD-PCR). For the amplification, the following primers were used (5′-3′ orientation):

TKTL1 fw: TTGTGTAGAGAAAGAAGATTTTG;
TKTL1 rev: ACCCCTTTAAAATCTAAAAACCC.

The sequence of the primer used for the amplification of the CpG islands was prepared with MethPrimer. After the PCR, the products were separated by agarose gel electrophoresis, the bands were isolated, purified by gel extraction, and used for sequencing. The same primers were used for the amplification of the CpG islands. The regions were sequenced with both the forward and reverse primers to avoid possible errors. 

For separation, the corresponding samples were mixed with a DNA loading buffer (Thermo Fisher, Karlsruhe, Germany) and then applied to the gel. The samples were separated on the gel for 90 min at a field strength of 3–10 V/cm, using 1xTAE as buffer solution. To visualize the DNA, 1x GelRed (Biotrend Chemikalien, Cologne, Germany) was added to the agarose gel before the gel was run and detected as a specific band pattern on gel documentation devices.

Touch-down PCR was used to amplify the TKTL1 promoter region after bisulfite conversion in the genomic DNA. Cycling conditions were as follows: annealing temperature at 64 °C for 5 min, reduction in temperature by 2 °C every single cycle, followed by 35 further cycles at 56 °C. To further increase the specificity, a hot start polymerase was used (EpiMark Hot Start Taq DNA Polymerase). For amplification the same primers as above were used. 

### 2.13. Statistics

Data are provided as mean ± standard error (SEM). All data were tested for significance using the independent Student *t*-test or ANOVA with Dunnett or Bonferroni correction. A *p*-value less than 0.05 was considered statistically significant. The statistical analyses were performed using GraphPad Prism 8.4.0 and SPSS software (version 26.0, IBM Corp. Armonk, NY, USA). To analyze the differences between TKTL1, Apo10, and combined TKTL1/Apo10 EDIM scores among RMS patients and healthy individuals, a receiver operating characteristics (ROC) analysis was performed and calculated using the Youden Index. To allow a sensitive and specific discrimination between cancer patients and healthy individuals, the ROC analysis was plotted to determine the best cut-off values of TKLT1, Apo10, and TKTL1/Apo10 EDIM scores in RMS patients compared to healthy controls. 

## 3. Results

### 3.1. Expression of Biomarkers TKTL1 and Apo10 in RMS Cell Lines

**Figure 1:**
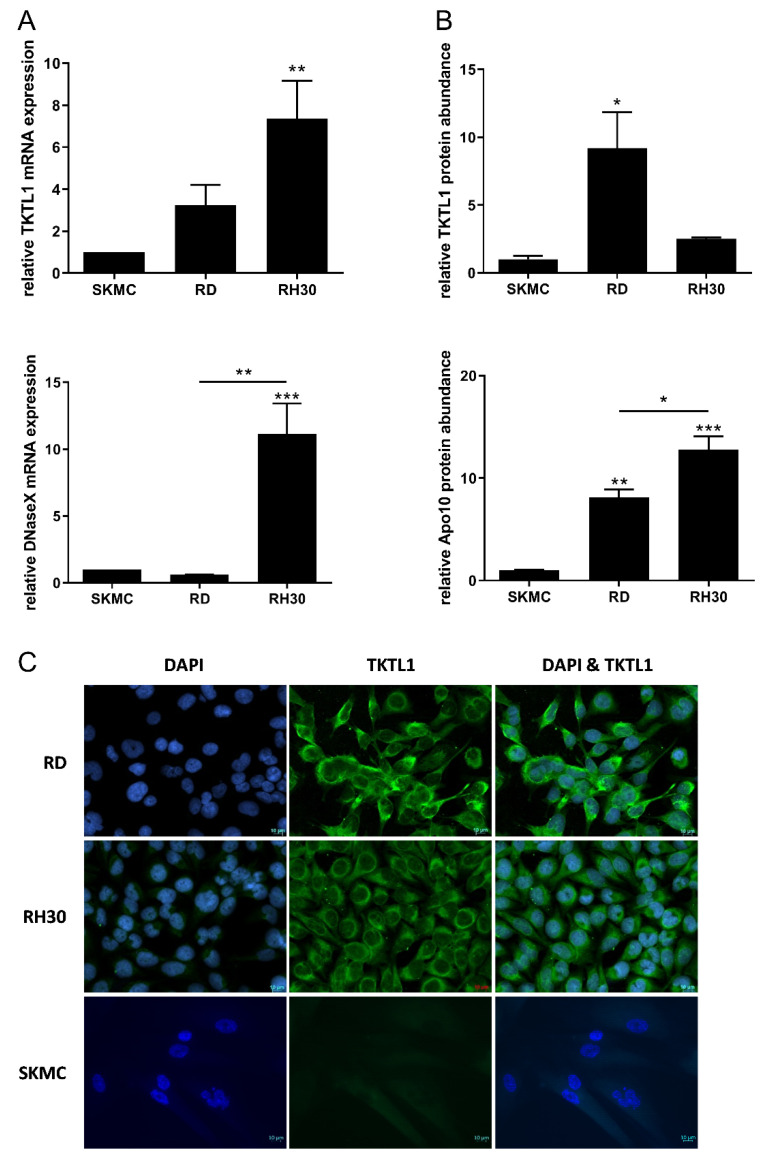
Expression of TKTL1 and DNaseX/Apo10 in RMS cell lines. Transcriptional expression of TKTL1 (upper graph) and DNaseX/Apo10 (lower graph) in the RMS cell lines RD and RH30, as well as in the SKMC cell line (**A**). TKTL1 and Apo10 protein abundance in RD, RH30, and SKMC cells (**B**). The expression levels in the figure are relative values. TKTL1 and Apo10 mRNA levels were measured by quantitative real time RT-PCR, with TBP as housekeeping gene. The data are presented as mean +/− standard error (SEM), *n* = 4; * *p* < 0.05; ** *p* < 0.01; *** *p* < 0.001 indicate statistical significance to SKMCs (ANOVA, Bonferroni correction). (**C**) Representative immunofluorescence staining and fluorescence microscopy of intracellular staining of TKTL1 (green) and DAPI for nuclei (blue) in RD, RH30, and SKMC cells. Original magnification, 40×.

The RD cell line showed a 3.2-fold higher expression of TKTL1-mRNA, while the RH30 cell line showed a 7.4-fold higher expression of the same biomarker compared to the SkMC. The expression level of TKTL1-mRNA in RD cells compared to RH30 cells was lower, however, only a trend toward increased expression of TKTL1-mRNA in RH30 cells could be observed (Figure 1A). 

The expression of DNaseX/Apo10-mRNA in RD cells was similar to that found in SkMC, while the RH30 cells had an 11.2-fold higher expression of the same biomarker compared to the control. The DNaseX/Apo10-mRNA expression was significantly higher in RH30 cells than in RD cells (Figure 1A). 

On the protein level (Figure 1B), TKTL1 abundance in RD cells was 9.1-fold higher than in SkMC, while the RH30 cells showed a 2.5-fold increased abundance of the TKTL1 protein compared to SkMC. The abundance of the epitope Apo10 was 8.1-fold higher in RD cells than in SkMCs (Figure 1B). In RH30 cells, significantly higher expression of Apo10 compared to SkMC was observed (Figure 1B). 

Fluorescence microscopy showed that TKTL1 was expressed in the cytoplasm of RD and RH30 cells. No expression of TKTL1 could detected in SKMC cells (Figure 1C).

### 3.2. Expression of Biomarkers TKTL1 and Apo10 in Tumor Material from Patients

**Figure 2:**
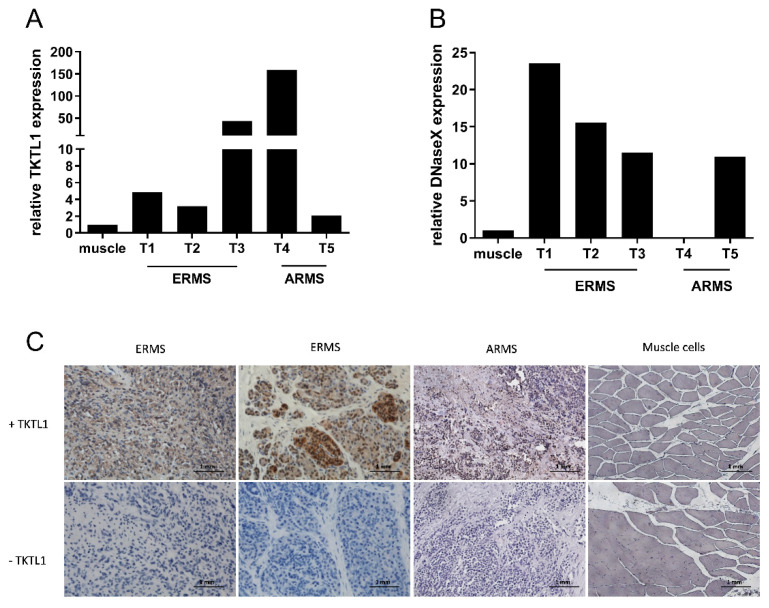
Expression of TKTL1 and DNaseX/Apo10 in tumor material from the patients. Transcriptional expression of TKTL1 (**A**) and Apo10 (**B**) in tissue samples from patients with RMS. Quantitative RT-PCR was performed to quantify mRNA levels, with TBP as a housekeeping gene. The expression levels are relative values (patient samples were compared to the respective control muscle tissues). ARMS: alveolar rhabdomyosarcoma; ERMS: embryonal rhabdomyosarcoma; Tx: number of the tumor sample. (**C**) Hematoxylin and eosin staining (control) and TKTL1 staining of ERMS, ARMS, and muscle tissue paraffin sections. Original magnification, 20×.

TKTL1 expression was up to 150-fold higher in tumor samples compared to normal muscle tissue, as shown in Figure 2A. The expression of the epitope Apo10 on protein level was up to 22.5-fold higher in tumors compared to normal tissue (Figure 2B). In addition, immunohistochemical analysis of TKTL1 expression was performed in paraffin sections obtained from five primary RMS cases and muscle tissue. Intense nuclear staining of TKTL1 was observed in RMS tissues, whereas lower intensity staining of TKTL1 was present in regular adjacent vascular structures. No staining of TKTL1 could be detected in muscle tissue (Figure 2C).

### 3.3. Co-Culture Experiments with Whole Blood and RMS Cell Lines

**Figure 3:**
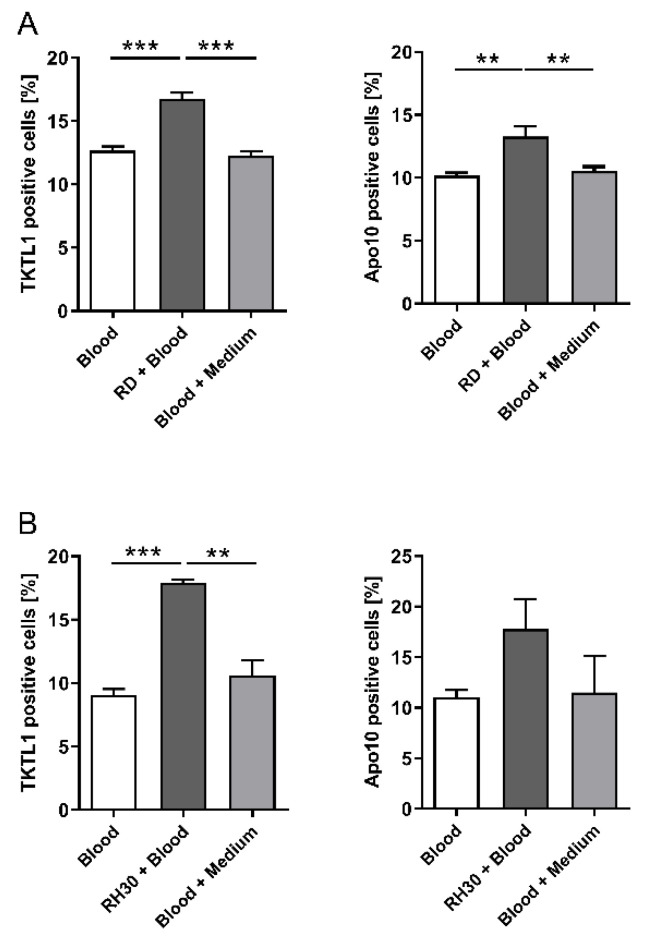
TKTL1 and Apo10 abundance in CD14^+^/CD16^+^ monocytes of co-cultured whole blood with RMS cells. The presence of TKTL1 and Apo10 in monocytes was measured after using the EDIM blood test via flow cytometry. Co-culture experiments with RD (**A**) and RH30 (**B**) cells show the uptake of the biomarkers in CD14^+^/CD16^+^ monocytes. White and grey bars indicate internal control with (grey) and without (white) medium. The data are presented as mean +/− standard error (SEM), *n* = 3; ** *p* < 0.01, *** *p* < 0.001 indicate statistical significance (ANOVA, Bonferroni correction).

To prove internalization of tumor particles by CD14^+^/CD16^+^ monocytes, co-culture experiments were performed. The incubation of peripheral blood with RD cells resulted in a significantly greater proportion of CD14^+^/CD16^+^ monocytes positive for both markers than for the controls (TKTL1: 17 % vs. 12 %, *p* < 0.01 and Apo10: 14 % vs. 10 %, *p* < 0.01), as shown in Figure 3A.

The incubation of peripheral blood with RH30 cells resulted in a significantly higher proportion of CD14^+^/CD16^+^ monocytes positive for TKTL1 compared to controls (17.5% vs. 10%, *p* < 0.001). The proportion of CD14^+^/CD16^+^ monocytes positive for Apo10 was also higher compared to controls (17.8% vs. 11.03%) (Figure 3B) and was even higher than the increase observed with the RD cell line. 

### 3.4. TKTL1 Expression and DNA-Hypomethylation in RMS Cells

**Figure 4:**
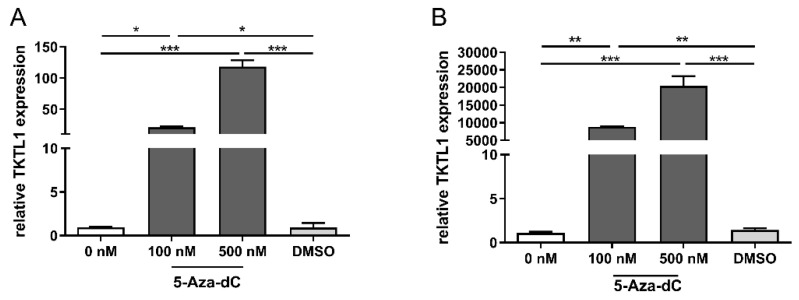
Relationship of TKTL1 expression at the degree of DNA methylation. TKTL1 expression of RD (**A**) and RH30 cell lines (**B**) after treatment with the DNA methyltransferase inhibitor 5-Aza-dC (decitabine). For the quantification of mRNA levels, a quantitative RT-PCR was performed using TBP as the internal control. The expression is given as relative value against to the untreated control. The data are presented as mean +/− standard error (SEM), *n* = 3; * *p* < 0.05, ** *p* < 0.01, *** *p* < 0.001 indicate statistical significance (ANOVA, Bonferroni correction).

The results of the quantitative RT-PCR showed an increase in TKTL1 expression in both cell lines after treatment with increasing 5-Aza-dC concentrations (Figure 4). Incubation of the cells with the same amount of DMSO (control) as the highest 5-Aza-dC concentration was not followed by an increase in TKTL1 expression. We observed that in those cell lines (RD) which usually had a lower TKTL1 expression (Figure 1A) even without treatment, the exposure to 5-Aza-dC led to a lower increase in expression, as shown in Figure 4A. In cells (RH30) that had a significant higher TKTL1 expression prior to treatment (Figure 1A), incubation with 5-Aza-dC led to a drastic enhanced expression (see Figure 4B). 

### 3.5. TKTL1 Expression and DNA Hypomethylation in Tumor Material

It has been previously reported that hypomethylation of the TKTL1 promoter leads to overexpression of TKTL1 in melanoma and squamous cell carcinoma of the head and neck [14,28]. Therefore, we investigated whether TKTL1 promoter hypomethylation is also a reason for TKTL1 activation in RMS. 

**Figure 5:**
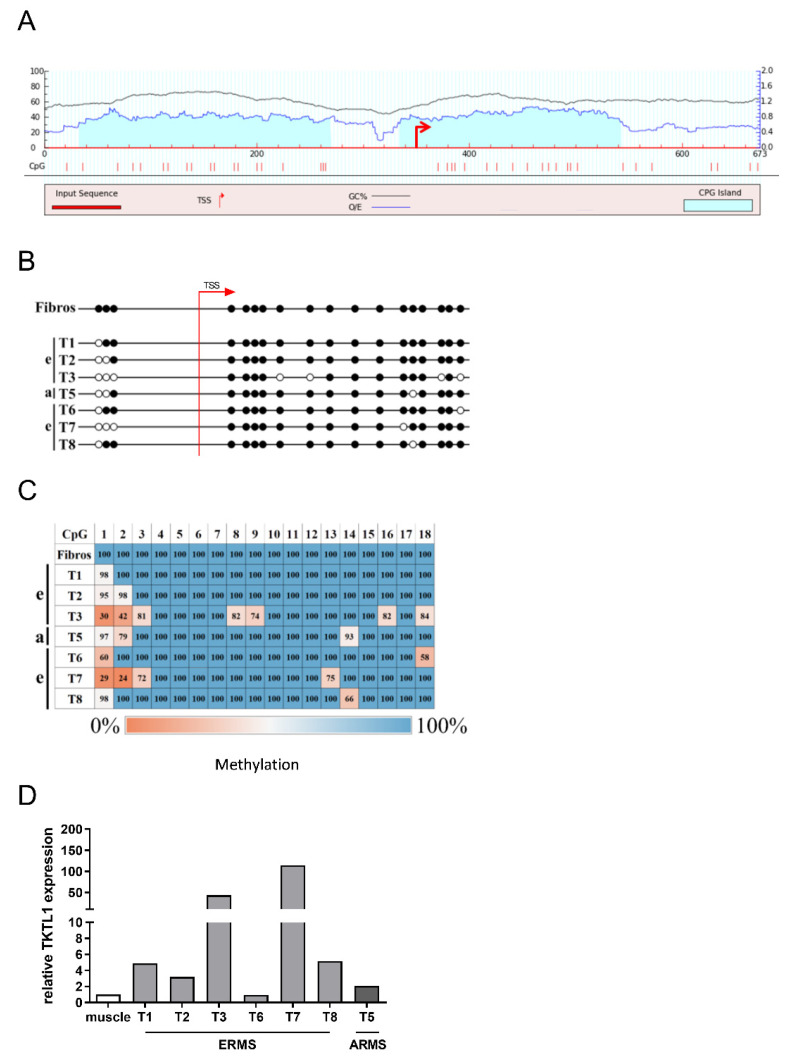
Semi-quantitative methylation analysis of a promoter region of TKTL1 in tumor material from patients with RMS. (**A**) In silico analysis to identify CpG islands in the TKTL1 promoter. Using the browser software MethPrimer, two CpG islands were found in the promoter of TKTL1. TSS and arrow: transcription initiation site; numbers are the size of the promotor in bp. (**B**) BS sequencing of the TKTL1 promoter region in RMS tissue samples. (**C**) Heatmap of the semi-quantitative methylation analysis of RMS tissue samples. The DNA methylation of each CpG dinucleotide is shown as percentage. (**D**) Relative expression of TKTL1 in RMS tissue samples used for quantitative methylation analysis. For the quantification of mRNA levels, a quantitative RT-PCR was performed, with TBP as internal control. The expression levels in the figure are relative values, as the patient samples were compared to respective control tissues. ARMS: alveolar rhabdomyosarcoma; ERMS: embryonal rhabdomyosarcoma; Tx: number of tumor sample; Fibros: fibroblasts; unfilled white circles: un-methylated CpGs; filled black circles: methylated CpGs. Note: As there was not sufficient tissue material available, three additional tissue samples (T6, T7, T8) were used for this experiment.

The results of the bisulfite sequencing showed that no un-methylated CpG dinucleotides were present in the CpG islands of the fibroblasts (control), as shown in Figure 5B. In contrast, all RMS tissue samples presented at least one hypomethylated CpG dinucleotide, as demonstrated in Figure 5C. Although additional un-methylated CpGs were present in 5/7 samples, these did not follow any pattern. Furthermore, they always occurred only in connection with hypomethylation of at least one of the first three CpGs. We observed that the first three CpGs belonged to the CpG island further upstream of the mainly investigated CpG islands.

Semi-quantitative DNA methylation analyses showed that in 4/7 RMS samples a very high degree of methylation was still present in the CpG dinucleotides (Figure 5C, T1, T2, T5, and T8). In these samples, the degree of methylation of the respective CpGs was above 90%. For T3, T6, and T7 samples, on the other hand, the CpG methylation rate was between 30% and 80%.

In order to investigate a correlation between DNA methylation of the TKTL1 promoter and TKTL1-mRNA expression, the expression analysis was also carried out with the abovementioned additional samples (Figure 5B,C). In the RMS samples, TKTL1 mRNA expression was the highest for tumor samples T3 and T7, which correlated with higher hypomethylation (Figure 5D). Only T6 was an exception, since un-methylated CpGs were found here, but TKTL1-mRNA expression was comparable to that of the control. 

### 3.6. EDIM Scores in Patients with RMS and Healthy Controls

In total, 29 consecutive patients with RMS were included in this part of the study. There were 12 females and 17 males. The median age at measurement was 40 months (8–252 months). Overall, 5 patients had ARMS and 24 patients had ERMS. In total, 24 patients were below 10 years of age, while 5 patients were older than 10 years of age. In total, 11 patients had tumors smaller than 5 cm, whereas 18 patients had tumors larger than 5 cm. Patient’s characteristics are detailed in Table 1. In the control group, 27 patients (11 females and 16 males) with a median age of 156 months (60–204 months) were enrolled. 

**Figure 6:**
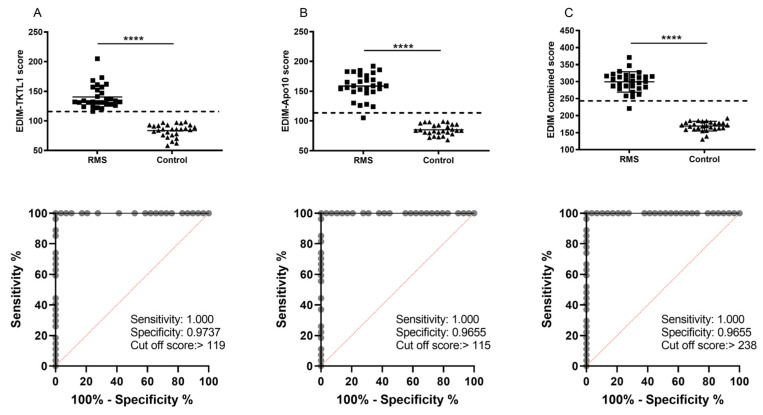
EDIM scores of the patients with rhabdomyosarcoma (RMS)**.** Receiver operating characteristics (ROC) analysis of TKTL1, Apo10, and combined TKTL1/Apo10 EDIM cut-off score in RMS patients compared with healthy individuals. The true positive rates (sensitivity) are plotted as a function of the false positive rate (1—specificity) for measuring the cut-off point; ROC analysis for the diagnosis of RMS shows calculated cut-off value with highest diagnostic accuracy of TKTL1 (cut-off score > 119; sensitivity 1.000, 95% CI 0.8754–1.000); specificity 0.9737, 95% CI 0.8282–0.9982), Apo10 (cut-off score > 115, sensitivity 1.000, 95% CI 0.8754–1.000; specificity 0.9655, 95% CI 0.8282–0.9982), and combined TKTL1/Apo10 score (cut-off score > 238; sensitivity 1.000, 95% CI 0.8754–1.000; specificity 0.9655, 95% CI 0.8282–0.9982). The dotted line shows the 95% CI. The scatter plots represent (**A**) the EDIM-TKTL1 score (cut-off > 119), (**B**) the EDIM-Apo10 score (cut-off > 115), and (**C**) the combined EDIM-TKTL1/Apo10 score (cut-off > 238). The dotted lines indicate the cut-off values of the scores. **** *p* < 0.0001indicates statistical significance (Students *T*-Test).

Measurements were performed at the time of diagnosis in 8 patients, before surgery (after neoadjuvant chemotherapy but before tumor resection) in 20 patients and 1 week after tumor resection in 1 patient. In those patients who received neoadjuvant chemotherapy before measurement, the time interval between blood sampling and the last chemotherapy block was 4 weeks. The results of the measurements are shown in Figure 6. 

EDIM-TKTL1 score was positive in 28/29 (96.5%) patients. EDIM-Apo10 score was positive in 28/29 (96.5%) patients. The combined score EDIM-TKTL1/Apo10 was positive in 28/29 (96.5%) patients. The calculated cut-off values with highest diagnostic accuracy of TKTL1 (cut-off score > 119; sensitivity 1.000, 95% CI 0.8754–1.000); specificity 0.9737, 95% CI 0.8282–0.9982), Apo10 (cut-off score > 115, sensitivity 1.000, 95% CI 0.8754–1.000; specificity 0.9655, 95% CI 0.8282–0.9982), and combined TKTL1/Apo10 score (cut-off score > 238; sensitivity 1.000, 95% CI 0.8754–1.000; specificity 0.9655, 95% CI 0.8282–0.9982) are depicted in Figure 6.

In RMS patients, the mean scores of EDIM-TKTL1 (140.4 ± 3.6), EDIM-Apo10 (158.6 ± 3.9), and EDIM-TKTL1/Apo10 (299 ± 5.6) were significantly higher than the corresponding scores in healthy patients (83.7 ± 2.1, 85.3 ± 1.7, 169 ± 2.7), respectively. 

In RMS patients, there was no significant difference of the EDIM-TKTL1, Apo10, and combined TKTL1/Apo10 mean scores depending on histological subtype, IRS stage, tumor size, patient’s age, and tumor location, as shown in Table 2. 

## 4. Discussion

The present study investigated the expression of biomarkers TKTL1 and DNaseX/Apo10 in RMS cell lines, in tumor samples as well as in blood samples taken from patients with RMS (using the EDIM blood test). Our results show that the embryonal cell line RD, as well as the alveolar cell line RH30, had a significantly higher expression level of TKTL1-mRNA and TKTL1-protein compared to the normal skeletal muscle cells (SkMC). The analysis of tumor material from patients confirmed these findings, as the expression of TKTL1 was up to 150-fold higher in RMS tumor samples compared to normal muscle tissue. Furthermore, we demonstrated for the first time by co-cultivation of RMS cells and blood with the CD14^+^/CD16^+^ monocytes contained therein, that there is a significant increase in the markers TKTL1 and Apo10 in CD14^+^/CD16^+^ monocytes. These results confirmed the previous hypothesis that CD14^+^/CD16^+^ monocytes phagocytize tumor cells and that the epitopes contained in CD14^+^/CD16^+^ monocytes can be detected by flow cytometry. Additionally, these data are of significant importance, as the basic principle of the EDIM technology, namely the phagocytosis of the tumor cells by CD14^+^/CD16^+^ monocytes and the subsequent detection of epitopes in CD14^+^/CD16^+^ monocytes by flow cytometry, has thus been demonstrated. The co-cultivation results also support previous studies in which other epitopes/proteins, such as prostate specific antigen (PSA) and carcinoembryonic antigen (CEA), were detected in CD14^+^/CD16^+^ monocytes [8,29]. These data demonstrate that EDIM technology is a new method to analyze the proteome of tumor cells in a non-invasive way through a blood sample. 

TKTL1 is a key enzyme in the non-oxidative arm of the PPP leading to increased lactate production, acidification of the tumor microenvironment with the destruction of the stroma, thus promoting cell migration and metastasis [15]. This has been observed in human colon cancer cells, where inhibition of TKTL1 gene expression has led to a reduced proliferation rate and decreased glucose metabolization [12,30]. Therefore, the high expression of TKTL1 may be regarded as a marker for uncontrolled cell proliferation and carcinogenesis [11,12,30]. It has also been reported that upregulation of TKTL1 is associated with poor prognosis in patients with certain type of malignancies, such as colon, urothelial, rectum, and laryngeal cancers [18,19,31,32]. In our patients we found no significant difference of the EDIM-TKTL1 and EDIM-Apo10 score depending on IRS stage. This aspect may be explained by the presence of a saturation effect, which means that a limited number of CD14^+^/CD16^+^ monocytes can phagocytize TKTL1 and Apo10 expressed by the tumor cells in patients with advanced stage disease [33]. Another possible explanation is that patients in advanced stages of disease receive intensified chemotherapy, which may result in a decrease in number of CD14^+^/CD16^+^ monocytes capable to phagocyte these biomarkers.

It should be noted that protein abundance does not always correlate with mRNA expression. A possible explanation is the stability of mRNA, a difference in the UTRs of the mRNA, or micro-RNA present in certain cells. Other processes that play important roles in the transcription or regulation of translation may also be involved. On the other side, quantification of expression in tumor cells and tumor material from the patient are relative values and are always given in comparison to control tissue or control cells. Additionally, comparisons between tumor cells and tumor material should be interpreted with caution, due to the tumor heterogeneity, the cells lines can never completely reflect a human disease [34]. 

The increased expression of TKTL1 in RMS cell lines may be related to hypomethylation of the TKTL1 promoter. DNA methylation, and in particular the methylation of CpG dinucleotides, represents some of the best known epigenetic mechanisms for gene regulation [35]. DNA hypomethylation has been proposed to play a role in cancer gene expression activation and genomic instability [28,36]. Rare examples of hypomethylation leading to activation of genes that are important in cancer have resulted in the overexpression of Bcl-2 and R-ras, resulting in the inhibition of apoptosis of tumor cells [28,37,38]. By evaluating the TKTL1 expression at the transcriptional and protein level, Smith et al. observed that TKTL1 was significantly overexpressed in head and neck squamous cell cancer cells in comparison to that in normal tissues [13,28]. In the TKTL1 methylated human oral keratinocyte cell line, they also reported transcriptional upregulation of TKTL1 after 5-Aza-dC, supporting the concordance between methylation and expression [13]. 

In our study, the treatment of RMS cells with the demethylation substance 5-Aza-dC resulted in a significantly increased TKTL1-mRNA expression. Additionally, we observed that RH30 cells (which usually presented a high expression of TKTL1-mRNA) after treatment with decitabine (an agent that inhibits DNA methylation) presented a dramatic upregulation of TKTL1-mRNA expression. 

In order to elucidate the mechanism behind the increased mRNA expression in RMS tumors, we analyzed the DNA hypomethylation of the TKTL1 gene. We observed that mostly three CpG dinucleotides (−157/158 bp, −159/160 bp, and −161/162 bp before transcription start) were affected. By additional mRNA expression analyses of the same tumor samples, an inverse correlation between methylation of this region and mRNA expression could be observed. Interestingly, the semi-quantitative methylation analysis showed that even in the samples with the highest TKTL1 mRNA expression, no completely hypomethylated CpGs were found. This heterogeneity of methylation could be an indication of a mixed tumor cell population (tumor heterogeneity), as it is often observed in different tumor entities [39] and might explain the different TKTL1 expression profiles in ARMS compared to RME. To our knowledge, this is the first study in the literature addressing the relationship between DNA hypomethylation and TKTL1 expression in pediatric RMS. However, further studies on a larger population are required.

Another protein which is overexpressed in many tumors is DNaseX. In tumor cells, the activity of this enzyme is blocked, resulting in uncontrolled cell proliferation and accumulation of DNaseX in the nucleus [10,11]. The monoclonal antibody Apo10 is used to identify the Apo10 epitope on DNaseX. Several authors reported on elevated epitope Apo10 in oral squamous cell carcinoma, lung carcinoma, breast carcinoma, and hepatoblastoma cell lines [10,12,23,40]. Our data confirmed these findings for RMS. We have observed that the expression of DNaseX/Apo10-mRNA was significantly higher in alveolar RH30 cells compared to normal cells, while the RD cells showed an expression of DNaseX/Apo10-mRNA similar to that of SkMCs. However, on the protein level, expression of Apo10 was significantly higher in both RD and RH30 cells compared to SkMCs. The analysis of tumor material from the patients confirmed these findings. We found that the expression of DNaseX/Apo10 was up to 22.5-fold higher in RMS tumor samples compared to normal muscle tissue. 

The present study demonstrated the diagnostic efficacy of the EDIM blood test in pediatric RMS. The TKTL1 and Apo10 scores were positive in 96.5% of the patients, respectively. All healthy individuals had a negative score of the corresponding biomarkers. This indicates a high specificity and a 100% sensitivity of the EDIM blood test. These results are in line with previously published results addressing other malignancies [6,12,41]. In a large cohort of patients with gastrointestinal cancer, Saman et al. found elevated TKTL1 and Apo10 scores in 95% and 89% of the patients, respectively. Grimm et al. also reported significantly increased TKTL1 and Apo10 scores in patients with oral squamous cell carcinoma compared with healthy individuals [6]. In their study, EDIM-Apo10 and EDIM-TKTL1 scores were positive in 92% and 93% of the patients, respectively [6]. We found no significant difference in the EDIM scores depending on histological subtype, age, and tumor size.

The data presented above suggest that liquid biopsy, in the future, may provide a non-invasive addition or alternative for tissue biopsy, imaging, therapeutic guidance, and monitoring of recurrence [24,42,43]. For example, Stegmaier et al. reported that liquid biopsy assay based on the detection of fusion transcripts in cell-free RNA from blood exosomes samples is suitable for analysis of patients with ARMS. Their results showed a good correlation with the initial tumor status, as liquid biopsy was positive in more than 90% of the patients with metastatic ARMS and initial bone marrow involvement, whereas biopsies from all patients with localized tumors were negative [24]. 

The present study has certain limitations. The first limitation is that, due to ethical reasons, only adult skeletal muscle cells (SkMC) could be used. Another potential weakness of the study resides in the lack of serial measurement of the scores in a large cohort. This fact may be explained by the rarity of this tumor. Furthermore, the amount of tumor compounds phagocytized by CD14^+^/CD16^+^ monocytes, could not be determined. Thus, the threshold of ingested tumor material necessary for determination as TKTL1 or Apo10 positive CD14^+^/CD16^+^ monocytes remains unclear at this point. The above-mentioned aspects, together with the correlation between EDIM scores and FDG-PET/CT/MRI imaging, should also be evaluated in further prospective studies. This might allow a preselection of cancer patients eligible for PET-CT/MRI imaging before, during, and after treatment. 

## 5. Conclusions

In conclusion, the RMS cell lines show an increased expression of the biomarkers TKTL1 and DNaseX/Apo10. DNA hypomethylation of TKTL1 promotor may play a role in upregulation of TKTL1 expression. The sensitivity and specificity of the EDIM blood test indicates that this new test might help improve the detection of pediatric RMS. Further studies on a larger population are required in order to critically assess the correlation between the test and clinical factors on serial measurements at diagnosis, during the therapy, and after completion of the therapy. 

## Figures and Tables

**Table 1:** Patient’s characteristics. Favorable localization: head and neck non-parameningeal, vagina, paratesticular; Unfavorable localization: extremities, trunk, bladder/prostate, perianal; IRS: Intergroup Rhabdomysarcoma Study; IRS I: macroscopically and microscopically complete tumor resection; IRS II: macroscopically complete tumor resection with microscopic residuals; IRS III: macroscopic residuals or biopsy.

Characteristics	Number of Patients *n* = 29 (%)
**Age**	
≥10 years	5 (17%)
<10 years	24 (83%)
**Sex**	
Female	12 (41%)
Male	17 (59%)
**Tumor localization**	
Favorable	3 (10%)
Unfavorable	26 (90%)
**Tumor size**	
≥5 cm	18 (62%)
<5 cm	11(38%)
**IRS Group**	
I	0 (0%)
II	1 (3%)
III	21 (72%)
IV	7 (25%)
**Histology**	
ARMS	5 (17%)
ERMS	24 (83%)

**Table 2:** EDIM scores depending on risk factors.

Variable	N	EDIM-TKTL1		EDIM-Apo10		EDIM-TKTL1/Apo10	
		Mean ± SE	*p*	Mean ± SE	*p*	Mean ± SE	*p*
**Histology**							
ARMS	5	135 ± 7	0.431	163.8 ± 10	0.952	299 ± 12.3	0.829
ERMS	24	141.6 ± 4.2		157.5 ± 4.3		299.1 ± 6.3	
**Age**							
<10 years	24	140 ± 4	0.887	159.4 ± 4.2	0.318	299.5 ± 5.8	0.172
≥10 years	5	141.8 ± 8.2		155 ± 11.6		296.8 ± 17.9	
**Size**							
<5 cm	11	141.8 ± 4.8	0.521	155.5 ± 6.5	0.659	297.36 ± 7.9	0.715
≥5 cm	18	139.6 ± 5.1		160.5 ± 4.9		300.1 ± 7.7	
**Localization**							
Favorable	3	128 ± 2.3	0.076	157.3 ± 15.3	0.772	285.3 ± 13	0.532
Unfavorable	26	141.8 ± 3.9		158.7 ± 4.1		300.6 ± 6	
**IRS Group**							
I–III	22	138.8 ± 3.4	0.089	157 ± 4.7	0.417	295.8 ± 6	0.622
IV	7	145.6 ± 11		163 ± 6.9		309.3 ± 13.5	

## Data Availability

Data can be made available upon reasonable request.
